# A Rare Variation of Transverse Testicular Ectopia (TTE) in a Young Adult as an Incidental Finding during Investigation for Testicular Pain

**DOI:** 10.1155/2018/6919387

**Published:** 2018-12-16

**Authors:** Chrysovalantis Gkekas, Evangelos N. Symeonidis, Ioannis Tsifountoudis, Christos Georgiadis, Vasileios Kalyvas, Apostolos Malioris, Michail Papathanasiou

**Affiliations:** ^1^Department of Urology, 424 General Military Hospital of Thessaloniki, Thessaloniki, Greece; ^2^Department of Radiology, 424 General Military Hospital of Thessaloniki, Thessaloniki, Greece

## Abstract

Transverse testicular ectopia (TTE) with fused vas deferens is an extremely rare clinical entity. Herein, we present a case of a 19-year-old patient with persistent left testicular pain lasting for a week. Clinical examination revealed an empty right hemiscrotum, a normal left-sided descended testis, and in close proximity a mass-like structure resembling testicular parenchyma. Laboratory tests were significant for elevated follicle-stimulating hormone (FSH), while sperm count revealed azoospermia. Ultrasound imaging (US) of the scrotum demonstrated the presence of both testes in the same left hemiscrotum with varicocele and no signs of inguinal hernia. Magnetic resonance imaging (MRI) of the penis and scrotum revealed TTE with a single, fused vas deferens, and hypoplastic seminal vesicles. Surgical intervention by means of microsurgical sperm retrieval and transseptal orchidopexy were considered but not performed, primarily owing to the patient's unwillingness and to a lesser extent due to the restriction that the short and fused vas would pose in an attempt to transpose the ectopic testis. Therefore, an annual follow-up was recommended.

## 1. Introduction

Transverse testicular ectopia (TTE) is a rare congenital anomaly in which both testes descend on the same inguinal route ultimately lying on the same side of the scrotum. This pathologic condition mostly affects young males with a mean age of 4 years. We present an interesting case of a young adult complaining of constant pain in the left testicle for 7 days. Clinical examination and imaging modalities established the diagnosis.

## 2. Case Presentation

A 19-year-old patient was referred to our department complaining of a week-long history of left testicular pain. His past medical history was remarkable for an absent right testis without any further clinical information. Upon physical examination, the patient presented with an empty and hypoplastic right hemiscrotum. The right testis was not palpable along the ipsilateral inguinal canal. On the contrary, a normal in size and consistency testis was present on the left side. Above that and in close proximity an oval-shaped smooth, parenchymal-like structure of considerably smaller size could be palpated.

**Figure 1:**
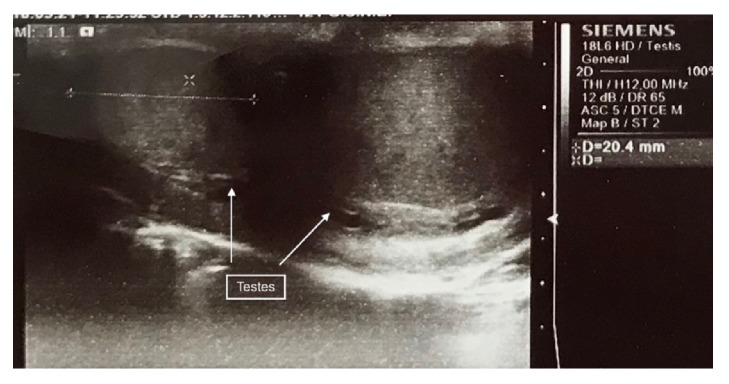
Sagittal ultrasound image depicting two testes in the same hemiscrotum.

The patient also complained of intermittent hypersensitivity over the left hemiscrotum, especially on prolonged standing. No signs of infection were present at the time. Clinically, a grade II varicocele with prominent dilated veins above the low lying testis could be detected upon Valsalva maneuver on the left side. Digital rectal examination revealed a small, nonpainful, normal feeling prostate. Laboratory workup was insignificant. Color Doppler US revealed the presence of two testes in the same left hemiscrotum and dilated pampiniform plexus veins. The caudal testis was of normal echogenicity, measuring 32x17x27.2 mm, and the cephalad one was comparatively smaller, approximately 20.4x17.8x11.2 mm, with similar echogenicity (Figure 1). Each testis was paired by a normal epididymis but only one vas deferens could be identified. The arterial supply of both testes was normal. Color Doppler study revealed a refluxing varicocele upon Valsalva maneuver at the lowermost part of the scrotum over the larger testis. There were no signs of an inguinal hernia sonographically.

Subsequently, the patient underwent upper and lower abdominal CT with contrast administration which revealed a normal urogenital tract but poorly visualized seminal vesicles. The CT findings excluded urogenital anomalies such as renal agenesis or malformation that are occasionally combined with testicular maldescent and ectopy. It also indirectly excluded the possibility of a right maldescended testis and a supernumerary left testis.

**Figure 2:**
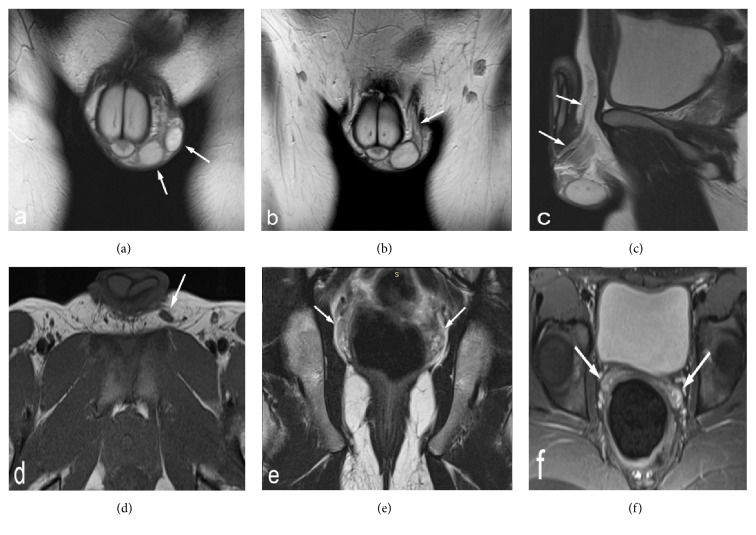
MR images of the scrotum and pelvis. (a) Coronal T2-w image reveals two testes in the left hemiscrotum, as ovoid-shaped masses with high signal intensity (arrows). (b, c, and d) Coronal T2-w, sagittal T2-w, and axial T1-w images demonstrate the single fused vas on the left side surrounded by fatty tissue (arrows). (e, f) Coronal T2-w and axial T2-w with fat suppression (FS) images depict the hypoplastic seminal vesicles with intermediate to high signal intensity (arrows).

An MRI of the pelvis and scrotum was additionally performed, which allowed to assess the seminal vesicles, the spermatic vasculature and the length and anatomy of the vas in further detail. The MRI findings demonstrated TTE with a single, fused vas deferens and bilaterally present but hypoplastic seminal vesicles (Figure 2). A sperm count revealed complete azoospermia and low ejaculate volume (1.3 ml). Blood tests showed a serum testosterone level of 8.5 ng ml^−1^, luteinizing hormone (LH) 7.9 mIU ml^−1^, alpha-fetoprotein (a-FP) 2.0 ng ml^−1^, beta-human chorionic gonadotropin (b-HCG) <1.2 mIU ml^−1^, and follicle-stimulating hormone (FSH) 26.22 mIU ml^−1^. Hormonal screening excluded androgen deficiency, but his elevated FSH implied primary testicular damage.

The patient refused any further investigation with testicular biopsy, as fertility was not his primary concern at the time. On the other hand, reconstruction and transseptal transfer of the ectopic testis to the contralateral side were deemed technically demanding, due to the presence of a single fused vas that would restrict testicular dissection and mobilization. Furthermore, the clinical benefit on his sperm count would be doubtful. Follow-up assessments were planned on a yearly basis with clinical examination, scrotal US, and hormonal screening since no orchidopexy was performed. This was an arbitrary follow-up scheme since there are no guideline recommendations or expert opinions in the literature.

## 3. Discussion

Testicular ectopia is an abnormally positioned testis that can lie anywhere along its theoretical descent route from the retroperitoneum down to the scrotum. In crossed or transverse ectopia the testis crosses the midline to land on the contralateral hemiscrotum [1–3]. This pathologic condition is thoroughly described in children, with a mean age of 4 years at the time of diagnosis, but rarely encountered in adults [1, 2, 4, 5]. It was first described in 1886 by Lenhossek on a 35-year-old adult [6]. Associated clinical findings most commonly include ipsilateral inguinal hernia, hypospadias, pseudohermaphroditism, and scrotal abnormalities or it can even present as part of the persistent Mullerian Duct syndrome [7–9].

Based on the associated developmental anomalies, a classification system was proposed in 1982 replacing a former one that relied on the etiology of this pathologic entity [10, 11]. According to Gauderer et al., three types of transverse ectopia are recognised: Type I, which is associated with an inguinal hernia and accounts for 40-50% of the cases; Type II, which is accompanied by Mullerian duct remnants (30%); and type III (13-20%), which includes genitourinary anomalies other than persistent Mullerian duct such as hypospadias, pseudo-hermaphroditism, bifid scrotum, renal anomalies, seminal vesicle contralateral aplasia, and seminal vesicle cysts [11].

Despite the fact that the two testes, in most cases, hang over a separate and distinct vas deferens, it has been described the variation of a common vas either as a single duct or an early fusion of two separates. This means that the single vas either originates from a common mesonephric duct, or from two separate counterparts that fused early during development after the crossing over of one of the two to the contralateral side. Gray and Skandalakis postulated that in cases where two distinct vasa deferentia exist, the testes develop from two separate ipsilateral urogenital ridges and the crossing over occurs during testicular migration [12]. According to Kimura who reviewed 11 cases of TTE, there is no true ectopia with an abnormal descent of the testis, unless two separate vasa exist [13].

The investigation of crossed testicular ectopia includes transabdominal ultrasound, MRI of abdomen and pelvis and contrast-enhanced CT to look for associated anomalies [3, 5, 14]. The role of MRI in the diagnostic approach of this pathologic entity is fundamental, as it can reliably distinguish the presence of two separate from a single fused vas deferens. The rarity of our case lies in the synchronous presence of bilaterally hypoplastic seminal vesicles (SV), varicocele, and azoospermia, extending the presenting spectrum of type III testicular ectopia which currently includes SV aplasia and SV cystic malformation [7]. Moreover, in our case testicular discomfort was a nonspecific, chance symptom that initiated the investigation and brought this condition to medical attention.

To our knowledge, Akin M et al. and Yıldız A et al. have reported the two largest case-series of TTE so far, each one with six patients [1, 15]. Most cases of TTE described in the literature are diagnosed before the age of 18 and management is targeted to protect fertility and reconstruct a normal anatomy by transferring the testis and repairing any associated anomalies such as inguinal hernias. Contrary to that, our case concerns a young adult without hernia. Gaur et al. reported a successful transseptal orchidopexy in a 21-year-old azoospermic adult with two equal-sized fused testes but with two distinct separate spermatic cords and normal vas [16]. Yanaral et al. did not perform a surgical correction in a 19-year-old azoospermic patient with fusion [3]. In 2015, Bascuna et al. proposed a treatment algorithm which included extensive mobilization of the vas and the spermatic vessels to allow for transseptal fixation [17]. This was subsequently challenged by Raj et al. in 2017 who argued for a less aggressive approach taking into consideration the length of the vas and even settling with a fixation in the same hemiscrotum without jeopardizing striping the testis off its vascular supply [18].

In any case the aforementioned algorithm applied to young patients, whose testes were still developing and could be favored by a reconstruction. In our case, the patient was an adult without associated anomalies to be treated and although we considered repairing the varicocele, the patient did not opt for concomitant varicocelectomy and testicular transfer for two reasons: firstly, because the fused vas would necessitate excessive dissection and mobilization of its common trunk distorting the anatomy of the native testis; secondly, a paucity of data in the literature favouring such a management for restoring fertility in adults. Our patient also refused considering microTESE and cryopreservation at the time reserving this option for later on. Interestingly, it is uncertain whether TTE poses an independent risk factor for testicular malignancy but has been linked to seminomatous, nonseminomatous germ cell tumours, and teratomas in published literature [19, 20].

In summary, TTE is a rare congenital anomaly which should be included in the differential diagnosis of every adult patient with an absent testis and fertility problems. In this setting, physicians should maintain a high index of clinical suspicion in every adult with symptoms of persistent testicular pain. If diagnosed, a thorough investigation should be employed for associated anomalies, taking into consideration the wide spectrum of associated conditions. Finally, it should be noted that the current classification system does not discriminate between a solitary and two distinct vasa deferentia, which might radically affect the therapeutic approach applied on patients exactly like the one presented here.
